# Geografías del (des)amparo: Bosquejos en salud mental antirracista

**DOI:** 10.18294/sc.2024.4890

**Published:** 2024-10-09

**Authors:** Jeannette del Carmen Tineo Durán

**Affiliations:** 1 Psicóloga Clínica. Estudiante Programa de doctorado en estudios interdisciplinares de género, Universidad Rey Juan Carlos, Madrid, España. jeannette.tineo@gmail.com Universidad Rey Juan Carlos Universidad Rey Juan Carlos Madrid Spain jeannette.tineo@gmail.com

**Keywords:** Salud Mental, Salud Comunitaria, Antirracismo, Migrantes, Mental Health, Community Health, Antiracism, Migrants

## Abstract

El presente ensayo explora los mapas afectivos o archivos emocionales de comunidades racializadas en España, en concreto, de la afrodiáspora caribeña en Madrid. Cuestiona cómo se prescribe gubernamentalmente el duelo migrante sin contar con la herida colonial, el trauma racial y las geopolíticas de las emociones, y ahonda en el racismo estructural cotidiano. Partiendo de la teoría decolonial y el feminismo negro, así como de las prácticas narrativas en sanación creadas por colectivos migrantes, se realizó una investigación cualitativa, en el período 2023-2024, en la que se realizaron 25 entrevistas en profundidad y dos talleres grupales con la participación de 15 personas activistas antirracistas. Tras una introducción desde una narrativa autobiográfica en la conversación antirracista, se señalan aspectos claves de la noción duelo migrante, asociándola con la herida colonial. Posteriormente, se enfatizan algunas características de la memoria como geografías del desamparo. Por último, apelo a la noción de imaginación, en modo de ensoñaciones o fantasías colectivas que activan la esperanza de traspasar las fronteras del racismo europeo. El artículo crea puentes entre la salud comunitaria, la perspectiva intercultural y el antirracismo.

## Abstract (en)

This essay explores the affective maps or emotional archives of racialized communities in Spain, specifically focusing on the Caribbean Afro-diaspora in Madrid. It questions how migratory grief is prescribed by the government without taking into account the colonial wound, racial trauma, and the geopolitics of emotions, while delving into everyday structural racism. Drawing from decolonial theory and Black feminism, as well as narrative healing practices created by migrant collectives, qualitative research was conducted during the period 2023-2024, involving 25 in-depth interviews and two group workshops with the participation of 15 anti-racist activists. After an introduction framed by an autobiographical narrative within the anti-racist conversation, key aspects of the notion of migratory grief are highlighted, associating it with the colonial wound. Subsequently, some characteristics of memory as geographies of abandonment are emphasized. Finally, I turn to the notion of imagination, in the form of collective daydreams or fantasies that activate the hope of transcending the borders of European racism. The article builds bridges between community health, an intercultural perspective, and anti-racism.

## INTRODUCCIÓN

“*Todo es diferente cuando estás lejos, intentas hablar por WhatsApp o Instagram, pero no es lo mismo, no coinciden las horas. Las videollamadas se quedan cortas, poco a poco te vas olvidando y en vez de hablar una vez al mes, es una vez cada 6 meses, una vez al año y poco a poco ya no recuerdas. [...] vas y vuelves [...] y ves las cosas de diferentes modos. Ves el antihaitianismo, el racismo, te ven blanca, te dicen así, cuando aquí nunca lo he sido [...] Y sientes por dentro que las palabras queman. ¿Digo algo al respecto? ¿No digo nada? ¿Tengo derecho a opinar cuando vengo de fuera? ¿Estoy en posición de señalar esos tipos de comportamientos cuando ni siquiera vivo allí? Lo veo desde unos ojos que han experimentado otro tipo de racismo, ya que aquí en su mayoría procede de gente blanca. Y allá es la experiencia del colorismo metida en la (no) negritud, porque según el muchacho del patio que me gritó rubia, allí soy ¡¿“blanca”?! Pero aquí yo soy negra*. (J. L. Entrevista en profundidad, 28 de febrero 2024)

La narrativa de J. L. refleja una resonancia constante con los pactos coloniales “allá y aquí”. En este ensayo me interesa señalar esa relación inseparable entre herida colonial, duelo migrante y trauma racial, con especial mención a lo que denomino “memorias del desamparo”. Para ello, se retoman aspectos claves de la ontología andina y las epistemologías feministas negras. Estos aspectos se omiten en el diseño y aplicación de las políticas migratorias, dado que estas responden a formatos de adoctrinamiento “del buen salvaje”, reforzando así el *anti-blacknes* del proyecto europeo. Los supuestos de la integración social están formulados para la consolidación de una fenomenología del espíritu colonial, en la que la introyección de los valores coloniales determina la psicopatología de la vida cotidiana^1^, cuya finalidad es sembrar el olvido. Por eso, el racismo estructural simbólico es traumatizante, pretende borrar aquello que funda la vida-muerte. En esa dialéctica de develar-negar lo ocurrido que señala Herman^2^, ocurre la *magia-arte-sanación* antirracista. 

Es necesario comprender las múltiples imbricaciones que abarca el proyecto migratorio para las personas no europeas, más aún la existencia transfronteriza^3^ en su devenir entre “aquí y allá” o en el marco de “chuzar el charco” transoceánico. Las migraciones, como fenómeno atravesado por las cuestiones de la racialización^4^, más aún como una expresión que atañe a los procedimientos de la colonialidad del género^5^, siguen siendo una cuestión pendiente en los estudios disponibles, dentro de lo denominado como cartografías de la diáspora^6^. Los estudios consultados centran su atención en la participación política, el acceso a servicios públicos, las cuestiones ligadas al empleo, la vivienda, la educación, la salud, la convivencia, etc., denotando sobre todo las barreras para la denominada integración social. Este trabajo apela, en cambio, a los mapas narrativos^7^, a los archivos de sentimientos que señala Cvetkovich^8^^)^ para abordar el trauma en un sentido colectivo, un sentido de comunidad propia que permite explorar o resignificar los espacios de las identidades en diáspora.

En la primera parte del ensayo esbozo algunas ideas respecto a mi lugar en la faena migratoria. Enmarco esta reflexión como “autobiografía política” como parte del giro emocional descolonial en la frontera^9^. En términos de Espinosa^10^, pretendo hacer una “genealogía de la experiencia” para interpelar nuestras prácticas sanadoras. En la siguiente sesión, abordo conceptualizaciones del duelo migrante y su vínculo con el trauma racial o herida colonial. Posteriormente, señalo algunos elementos característicos de las tramas afectivas asociadas a las geografías del (des)amparo. Finalmente exploro algunas ideas acerca de la sanación (salud) comunitaria antirracista, como parte de conclusiones abiertas a la profundización. 

## ACERCA DE LA INVESTIGACIÓN

Lo que aquí se plantea forma parte de mi trabajo de tesis doctoral “Cartografías afectivas de la afrodiáspora caribeña en Madrid”. Una investigación con metodología cualitativa, en la que retomo elementos de las prácticas narrativas, el arte terapia y el análisis de discurso en ciencias sociales. Aplico la autoetnografía como forma de exponer mi/nuestro lugar de enunciación; y la etnografía afectiva, como parte de “escrituras afectivas que se convierten en praxis políticas que nos recuerdan que somos cuerpxs que encarnan historias que reclaman ser contadas”^11^. Se trata de una exploración inspirada en la biomitográfica propuesta por Audre Lorde^12^, basada en políticas del feminismo negro para renombrarnos. Esta estrategia consiste en mapeos del archivo del corazón, según vaivenes, epifanías o posmemorias de “mi autohistoria como eslabón colectivo”^13^. Se trata de prácticas investigativas para la resignificación de los territorios psíquicos*,* como instancias que preservan el material de la represión, negación, repetición y desplazamiento al que debe ajustarse la existencia racializada. 

Además del trabajo autoetnográfico, en la investigación se utilizaron otras herramientas cualitativas de “articulación comprometida”^14^ con la comunidad antirracista en Madrid, durante el período 2023-2024. Se realizaron 25 entrevistas a profundidad, dos talleres de reflexión sobre las epistemologías decoloniales y antirracistas en el contexto de la migración, y observación participante en escenarios como manifestaciones sociales, exposiciones artísticas, etc. Los talleres tuvieron un carácter experiencial, a modo de laboratorio de creación colectiva, en el que se pusieron en práctica herramientas del arte-terapia. En estos laboratorios se realizaron análisis del discursos de imágenes o formas de presentación colonial que circulan cotidianamente. 

Participaron de estas acciones metodológicas un total de 40 participantes jóvenes, de entre 20-35 años, y adultos, de entre 35-50 años, a quienes se accedió mediante la técnica “bola de nieve” (a través de los contactos con la comunidad antirracista de la que formo parte). Los criterios de selección fueron: haber vivido un proceso migratorio en Madrid, con procedencias y orígenes diversos en términos de la experiencia racial. En su mayoría se identificaron como afrodescendientes, disidentes sexuales y de género. Las entrevistas fueron grabadas en audio y transcritas para su análisis narrativo. Tanto en las entrevistas como en los talleres realizados se garantizaron los procedimientos de confidencialidad y protección de datos de acuerdo con las normativas vigentes. Se realizaron procedimientos de consentimiento informado para cada uno de los procedimientos metodológicos realizados y la investigación contó con la aprobación del comité de ética de la Universidad Rey Juan Carlos (número de registro interno 220120241382024). 

## LUGAR DE ENUNCIACIÓN

“Escribo para sobrevivir. Es un compromiso con las mujeres que vinieron antes de mí, con las que están aquí y con las que están por llegar. Si se queda dentro de mí, exploto”.^15^

Retomo el aporte de Ribeiro^16^ sobre cuán necesario es “romper con el régimen de autorización discursiva”, para refutar, sorprender y traspasar el funcionalismo de la universalización del ser mujer, lesbiana, caribeña, “afro clarita”, psicóloga, migrante entre otras identidades que me arropan o ahogan. Utilizo el ahogo como una metáfora que atraviesa mi cuerpo: teniendo 6 años casi muero intentando aprender a nadar, pero esa es otra historia. 

Mi nombre se escribe en francés y se pronuncia en español. Siempre me toca deletrearlo. Mi nombre habla de las fantasías blancas criollas ocultas o ensalzadas en la creación de dominicana. Una tierra nombrada como *“madre de todas las tierras”* por la cosmogonía Taina-Arahuaca antes de la colonización. A partir de ahí, en función de los intereses franceses y españoles, una tierra partida en cuatro fronteras con Haití que alimentan el rechazo de lo que somos en la negritud. 

Soy hija de las aguas dulces. Mi abuela me enseñó que, si estaba triste, el mejor remedio era zambullirme en el rio para que las penas se fueran. Hasta hoy es así para mí. Quizás por eso, aunque Madrid Rio es un chorrito de agua, me encanta sentarme ahí cuando las cosas se ponen difíciles aquí. También porque cerca de ahí está *Kukaramacara* con el mejor picapollo, ruido y bachata de aquí. También están los contornos del Matadero, el Ayllú, La Parcería, Conciencia Afro..., trenzados antirracistas que caminamos con (des)amor colectivo, creaciones de belleza a punta de la interrupción al arte blanco o, como dice Danticat^17^, “crear en peligro el trabajo del artista migrante”. Este autor señala algo contundente respecto de nuestras sensaciones más profundas: “creemos que somos los hijos de personas que han vivido en la sombra por mucho tiempo [...] creemos que somos un accidente [...] creamos como si cada pieza de arte fuera un sustituto de una vida, de un alma, de un futuro” ^17^.

**Figura 1:**
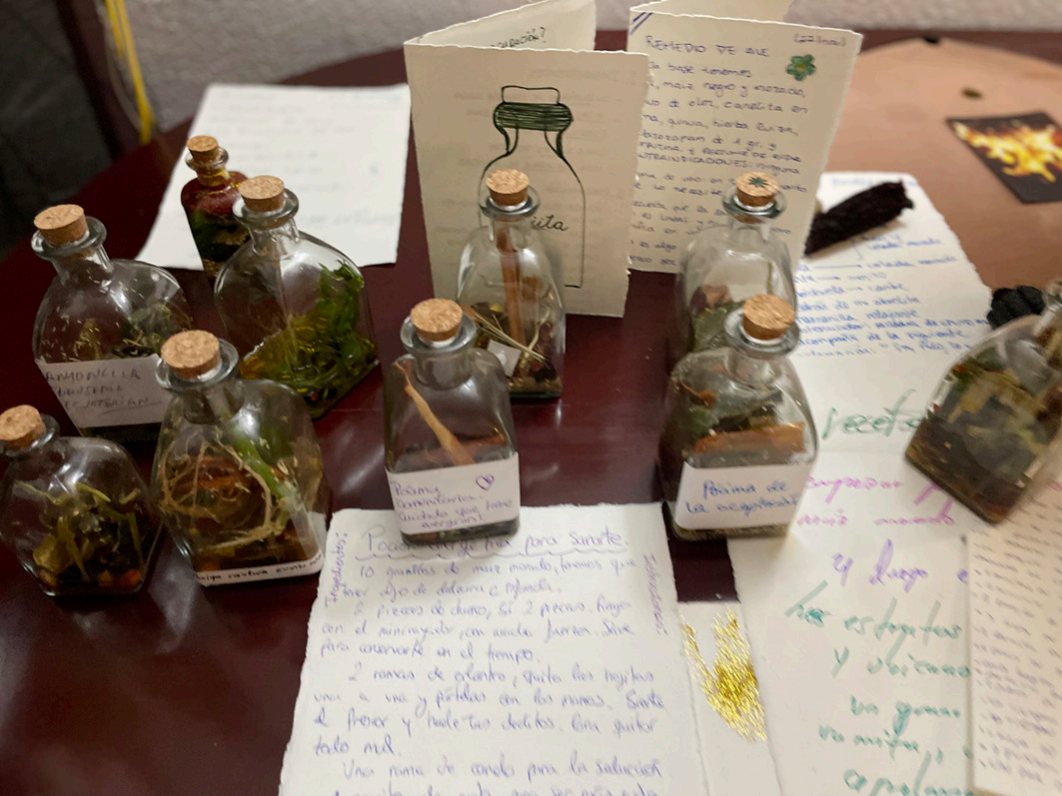
Pócimas anticoloniales.

Tras vivir una pandemia en Madrid y colectivizar pérdidas, (des)enamorarme y vuelta a enamorar (no solo románticamente), me doy cuenta de que quiero a Madrid. Y no en el modo del lema de la campaña “*ames a quien ames Madrid te quiere”* (World Pride, Madrid 2016)*,* sino que lo quiero en el modo marrón “morisco”, en el *morenaje* que destila Vallecas, donde vivo. Como un vino o ron con cuerpo. Este amor tampoco es al modo inicial cuando llegué, cuando las fantasías de cruzar el charco^18^ estaban a flor de clítoris lésbico, con mucho “bollo drama”^19^ trasatlántico, aprendí lo que no quería olvidar, pero tampoco repetir en mi existencia lésbica^20^. Quiero a Madrid a partir del hocico y el olfato de mi perra: Teresa (por Santa Teresa) que ya no está, sus cenizas las esparcí en su parque favorito, el Tierno Galván, entonces algo de mí ya está en Madrí. En fin, este texto como mi tesis es una escritura para recuperar el sentido de lugar que nos fue arrebato, por eso trata del “Caribe en Madri”^21^, con nuestras pócimas que nunca fueron colonizadas. Mi conciencia caribeña campesina se ha alimentado en la extranjería y en el intento de construir “hogar en la diáspora”; con el deseo de hogar que no es lo mismo que patria^22^. La Figura 1 es parte de esos procesos creativos de pócimas que imaginamos que acompañan nuestra diáspora en Madrid, realizados en los talleres-laboratorios explicados anteriormente. 

Fuente: Taller-Laboratorio creativo “Un hogar en la diáspora”. Residencia Felipa Manuela. Madrid, octubre 2023.

Nuestra sanación pasa por lugares que el sistema de sanidad desconoce. Por eso me interesan las cartografías afectivas, porque articulan otra curación narrativa. Permiten abrazar los “pedazos rotos” por siglos de colonización. Los mapas son andamiajes que permiten resignificar el lugar o los espacios. Contarnos sin ser víctimas en espera de salvadores. La primera lectura que me ayudó a articular las ideas de la geografía emocional fue años atrás Linda MacDowell^23^ y su articulación feminista en torno a la producción del género como cuestión dada por el lugar. También buscaba cómo traspasar el régimen de la heterosexualidad obligatoria^24^. En esas conversaciones de esquinas, camas, colmados, tomándonos la ciudad colonial en Santo Domingo, llegué a la conclusión de que necesitaba palabras que me permitieran decir que las sexualidades son geografías emocionales. Insistía en querer un “psicoanálisis tropical” como diría mi terapeuta. En esos términos honro las reflexiones de Suely Rolny^25^, Frank Fanon^26^ y Grada Kilomba^27^. 

Cuando terminé de estudiar psicoanálisis a finales de la década de 1990 comprendí que todo el bagaje de sanación afrocaribeña^28^ que caracteriza nuestros mundos fue omitido en mis procesos de aprendizaje de la psicoterapia. Lamenté y sufrí la desconexión que me generaban las filosofías francesas, alemanas e inglesas con las que aprendí a comprender “la mente”. Desde ahí empecé un largo recorrido, hasta hoy, para aplicar en la terapia metodologías de la carne, comunitarias o de la educación popular y la psicología de la liberación^29^. 

Definitivamente, mi imaginación caníbal=caribeña implica recordar que algo en mí se entreteje devorando. Eso pretendo: quitarme toda la blanquitud impuesta y habitar el mundo sin cargas ancestrales, sin la pasión por llegar a ser, gozosa, descansando y honrando lo que soy. En buen dominicano: “que no me jodan” mis fantasmas blancos y negros. Por todo eso y más, procuro comprender qué nos pasa, para mí esto siempre sobrepasó a un deseo teórico, siguiendo a Moraga^30^: “el peligro radica en tratar de enfrentar esta opresión en términos meramente teóricos. Sin una envoltura emocional sentida en el corazón que surja de nuestra opresión, sin que se nombre al enemigo que llevamos dentro de nosotras mismas”. Para eso es este texto, para contribuir al *depojo*, a la limpieza o sanación en el sentido de preservación afroindígena resguardadas en las prácticas de las 21 divisiones (sistemas de protección y aseguranza de la vida mediante prácticas o cosmogonías afroespirituales dominicanas). 

Este breve relato da cuenta de cómo he ido experimentando transformaciones que se entrelazan con los pasajes colectivos del duelo migrante, la herida colonial y las distintas prácticas que se generan para preservar memorias, genealogías y relatos antirracistas del sanar aquí en el lugar de diásporas que la sanidad no contempla. Esto es lo que exploro a continuación. 

### Duelo migrante: Pasajes por la herida colonial

Duelo migratorio^31^ se utiliza para describir el estrés crónico, el quebranto que genera el desarraigo en la situación migrante. Este concepto se ha popularizado en las políticas sociosanitarias y en el movimiento antirracista en España. Su uso universalista es una práctica europeizante que se compara con el “Síndrome de Ulises”. Esta alusión al mito griego refuerza imaginarios de la migración como estados blancos individuales en perenne soledad, sin reconocer los territorios de identidad transfronteriza que caracterizan las diásporas racializadas a lo largo de la historia. Dicho de otro modo, no se imbrica el duelo migrante a las cargas sistémicas inscritas en la memoria (anti)colonial. 

En las descripciones habituales del duelo migratorio se disuelve el racismo estructural, por tanto, el sufrimiento se concibe como parte de UN trauma único esencial con sintomatología que patologiza la existencia racializada. Se produce un borramiento en los sistemas sanitarios de los determinantes estructurales de la racialización superpuestos al género, la edad, la clase y la sexualidad entre otras intersecciones^32^. Ello implica que los abordajes en salud mental se reducen y no consideran la externalización de los problemas asociados a la raza^33^. En la experiencia siguiente se muestra esta omisión:

“*Lo más difícil para mí es llegar a la cita médica, después de meses incluso años de espera* [...] *Apenas abro la boca para explicar lo que siento, la angustia o el terror, que uno no pueda dormir, hay una interrupción para darte una receta, mi dolor no se resuelve solamente con lorazepam o sertralina* [...] *Además frente a una persona cis blanca elijo callarme porque sé que no va a comprender mi deseo de desaparecer, lo que pesa en mi cuerpo. Creo que ni notan mi dolor. Son incapaces de comprender todas mis partes. Creo que mi diferencia es tan notoria que optan por no verla*. (A. M. Entrevista en profundidad, 20 de febrero 2024).

El silenciamiento del racismo impacta gravemente en la salud mental y esto es crucial en las experiencias migrantes. En la siguiente definición se observa cómo se utiliza el concepto duelo migrante en términos de capacidades individuales del sujeto migrante sin valorar los contextos de racialización.

“El duelo migratorio se caracteriza por ser una situación que somete a la persona que migra a cambios múltiples y permanentes al mismo tiempo. Es decir, es un duelo que se mantiene activo durante toda la vida migratoria de la persona, estando muy relacionado con las vivencias en el país de origen y vinculado a las capacidades personales de adaptación y resiliencia”.^34^

La tendencia en las definiciones exploradas se relaciona con imaginarios supremacistas acerca de la identidad del “buen migrante”. Es decir, las tramas políticas de integración social europea procuran que las personas racializadas, a modo de tabula rasa, se articulen al proyecto de modernización^35^. En otras palabras, adoctrinar los cuerpos considerados bárbaros bajo supuestos de equidad en la atención es parte de la tecnología colonial que no reconoce las raíces del sufrimiento como parte del racismo estructural. Este procedimiento se articula en políticas liberales de integración social que procuran la asimilación, adaptación y demostración de que se poseen grados, rasgos o credenciales definitorias del espíritu civilizatorio para el ascenso a la blanquitud^36^.

Duelo migrante por sí solo es limitante. Es una categoría reduccionista en tanto no se vincula con la herida colonial. De este modo, la cultura política de las emociones^37^ migrantes deseables, se pretende ajustar a la prolongación de la supremacía blanca, dentro de las ideologías atomistas individualistas que caracterizan las intervenciones sociosanitarias^38^. 

El padecimiento emocional de las personas racializadas, siguiendo a Fanon^26^, ocurre en las zonas de no-ser pautado por la configuración de “objetos en medio de otros objetos”. Esta metáfora indica que el constreñimiento afectivo está condicionado por fronteras (no) visibles establecidas en marcos de la Unión Europea que indican cuáles vidas importan o cuáles no. 

El dolor migrante en términos de racialización, según la ontología andina, navega como parte del Pachakuti; es decir, con el cambio radical de los sentidos ocasionado por la colonización que disoció la relación con el todo, con las estructuras del “arriba” y “abajo”, creando un cambio cósmico violento^39^. Dicho de otro modo, en los términos de la tradición afrodiaspórica se trata de un duelo profundo, arraigado en las grietas infringidas en la memoria como parte del *maafa* o el holocausto ocasionado por la esclavización, de la trata transatlántica iniciada en 1492, continuada en las formas modernas coloniales de la migración. 

Es significativo asociar la experiencia de la racialización y el duelo migrante al holocausto, como metáfora física no exclusiva del pueblo judío, más bien como experiencia entretejida en la partición del mundo para la creación de Europa. Según Quijano^40^, este procedimiento de clasificación social, basado en la raza, se ha ido renovando y ajustando como colonialidad del poder. Desde esta idea, la modernidad de Europa, sus luces, progreso, ilustración, riqueza o bienestar oculta su lado genocida o sangriento: la continuidad del colonialismo, expresado en el maltrato a los sujetos coloniales globales en el contexto de la migración^41^. 

El duelo migrante como concepto europeo, producido en los contextos de afinamiento de las políticas sociales de la Unión Europea, es insuficiente desde una perspectiva antirracista. Su definición aplicada adolece de una posición reparadora en términos de memoria histórica que le permita coexistir a las personas racializadas, traspasando los contornos virulentos del proyecto colonial. Y, al contrario, las leyes de extranjería y su aplicación en los ámbitos sanitarios consolidan los discursos prácticos del racismo institucional. 

**Figura 2:**
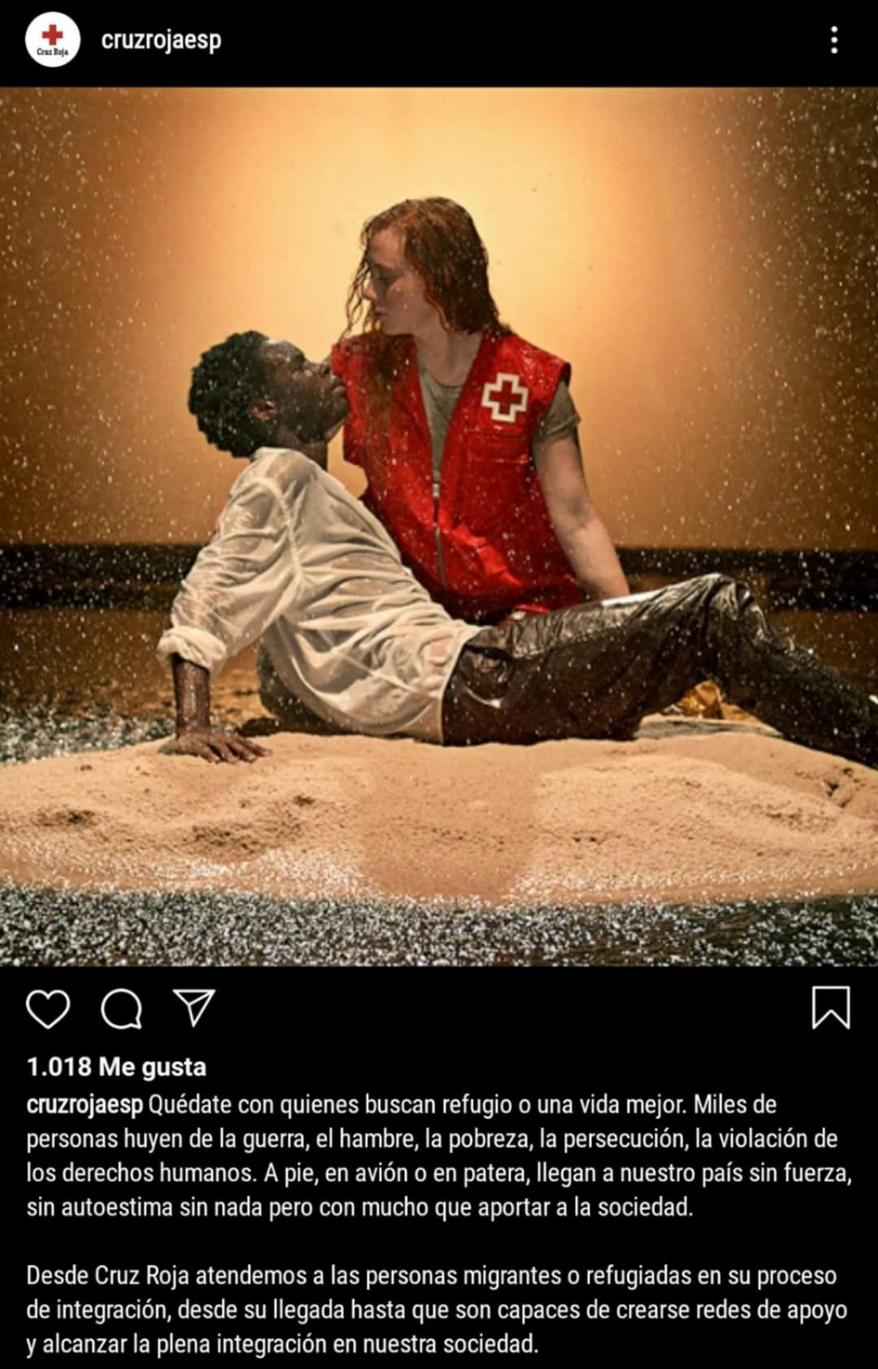
Quédate con quienes buscan refugio.

La instrumentalización del duelo migrante ocurre bajo formatos de salvación, caridad o culpa que pautan las relaciones de superioridad blanca, tal como se muestra en la Figura 2 (analizada durante los talleres). Las historias migrantes racializadas son operadas por políticas asistencialistas europeas ancladas en el imaginario salvador/víctima, reforzadas por los programas de acogida de protección social. “El síndrome de salvador o salvadora blanca es una práctica social que explota o saca provecho económico, político o simbólico de las personas o comunidades racializadas, que, a los ojos de la blanquitud, están salvando y deberían estar en deuda y en eterna gratitud”^42^. 

Fuente: Cruz Roja España. 13 nov 2019. @cruzrojaesp.

Este fenómeno trata de la colocación de los cuerpos racializados en posiciones de exterioridad sometida a la ayuda, a las “buenas intenciones” o condescendencia, típica de la caridad cristiana, reforzando así relaciones basadas en infantilización, codependencia y exotismo. Como se observa en la fotografía (Figura 2), los cuerpos migrantes racializados son seres suplicantes, deseosos del paternalismo europeo. En la imagen se amplifica el ideario colonial, en tanto se romantizan las relaciones raciales de género y el régimen de la cis heteronorma. A pesar de las más de 100 críticas recibidas en el *post,* la imagen no ha sido retirada de la web de Cruz Roja, demarcando el poder violento naturalizado de la arrogancia o inocencia blanca^43^. El descontento, la rabia y frustración por estas prácticas fue expresado en los siguientes términos:

“*Lo más difícil de nuestra vida aquí es que no toman en cuenta nuestras experiencias, nuestras capacidades, lo único que les importa a las instituciones, tanto las LGBTIQ como las de refugio, es llenar listados, utilizarnos para sus campañas en redes sociales, utilizar nuestro testimonio para vendernos para comercializar con nuestro sufrimiento* […] *Yo he pasado por todos esas ONG, por todos esos proyectos que supuestamente son para personas migrantes y LGBT, pero con todas me termino peleando porque no les gusta que mostremos nuestro liderazgo, no le gusta que pongamos nuestro saber, nos tratan como menores o problemáticas, su idea es hacer una fotografía, un video* […] *¿Por qué no nos dan trabajo? ¿Por qué mejor no nos contratan? Por qué, en vez de capacitarnos en cursos de maquillaje, de cocina, de peluquería, etc., y de llenar esas listas, por qué mejor no crean condiciones de trabajo. De seguir estudiando, trabajando. Yo todo lo que quiero es eso, no estoy pidiendo que me regalen nada. Y nos tratan así, nos dan comida, nos dan un bono para el transporte, o cosas de esas, pero eso no es lo que necesitamos. Necesitamos trabajos que lo único que nos queda a las trans es la calle, el desamparo y las violencias que se viven ahí por parte de la misma policía es grave para nosotras* […] *Estoy harta* […] *A mí lo que me ha salvado es encontrarme con una comunidad de gente joven disidentes que bailan, que son artistas, que hacen cosas maravillosas y aunque no tenemos dinero, al menos sabemos que somos como una familia*. (F. H. Entrevista en profundidad, 12 de enero de 2024)

El duelo migrante está concatenado a las esferas de la dominación colonial, a las demandas de una subjetividad anclada en la dependencia. Por tanto, su tratamiento no puede asumirse en términos de una patología o síndrome, se trata de una experiencia colectiva de dolor que no puede mitigarse con el uso de diazepam. La resolución encuentra asidero en la activación de comunidades que sanan en la diáspora. En términos de Iki Yos Narváez^44^, la diáspora es un lugar que se rehace, “que no termina” según “comunicaciones telepáticas o *tecnoafectividades* que degeneran la razón europea”, se relaciona con “malungaje, con la desobediencia que atraviesa el océano”^44^. 

**Figura 3:**
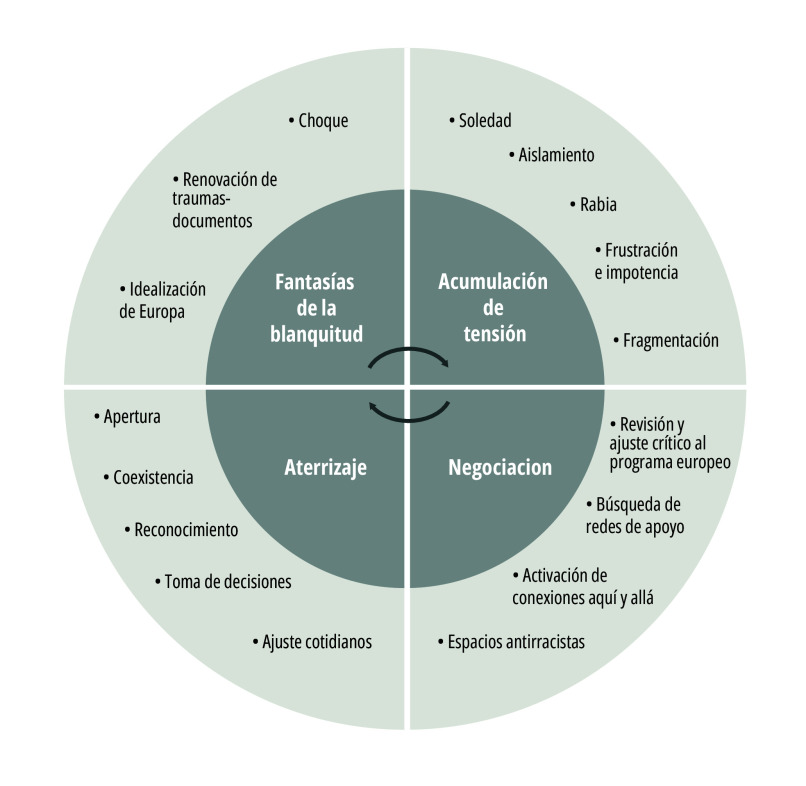
Duelo migrante y racialización.

La Figura 3 muestra las emociones identificadas acerca del duelo migrante racializado, las cuales están imbricadas a las fantasías o promesas europeas y los imaginarios de lugar asociados al “retraso” del Sur Global. Este conjunto de emociones, desde la teoría del apego y su relación con traumas de pérdidas^45^, indica que las personas racializadas devienen en tramas psicosociales pautadas por la evitación, ambigüedad y ansiedad con escasos andamios para el arraigo, la vinculación segura o sentido de pertenencia. Los trámites de extranjería se encargan de sostener esa posición trapecista de los cuerpos, sobre todo para quienes “no tienen papeles”. La burocracia migratoria está diseñada para no permitir respirar, para mantener el cuerpo en vigilancia y zozobra. La pregunta constante, como detonante o gatillo del malestar es: “¿de dónde eres?”. 

Fuente: Elaboración propia.

Oficialmente, el duelo migrante se define como recurrente, como proceso abierto que nunca cierra. Este abordaje es estratégico a los intereses europeizantes. Permite que en Europa nunca reclamemos tierra o reparación histórica, que nuestros aportes en términos de economías afectivas-dinero, tanto en lugares de origen como aquí, queden en el borrado-olvido. Pareciera que la identidad está sujeta al *“ni de aquí ni de allá”* y esa convención facilita los proyectos de acumulación (tensión) del capital racial europeo. Si bien desarrollamos una subjetividad “en medio”, “entre aquí-allá”, de experimentación simultanea del espacio-tiempo, en términos de sanación colectiva es poderoso el pronunciamiento “me fui a volver”^46^, porque nos permitimos vivir (sin-con-desde) las fronteras. 

El contrato colonial se lleva en la espalda^47^, como montura poblada de culpas, miedos, abusos, lealdades sistémicas, que pesan mucho más que los 23 kilos que permiten las aerolíneas para viajar. Se trata de resistencias cotidianas “aquí y allá” como, por ejemplo, “La Manta” que dignamente portan los manteros en la Gran Vía, mal tratados por el racismo contemporáneo en la *ratio animalitas* de la metafísica occidental que describe Mbembe^48^, resumida en brutalidad policial. Son estos actos, junto a otras formas de violencia racial, los que no pueden omitirse en la atención al duelo migrante. 

La obviedad de que las migraciones están cruzadas por la racialización no es notoria en las propuestas de políticas públicas, más bien es una materialidad que se objeta. Por esto, duelo migrante se convierte en un concepto descafeinado que incluye las experiencias de viajes de personas blancas o blanco mestizas que poco tienen que ver con el trato asentado en la necropolítica europea^49^. En el siguiente apartado profundizo está cuestión como memorias que aprietan el (des)amparo. 

### Trauma racial y memorias del (des)amparo

Trauma racial refiere a convivir sistemáticamente las disparidades generadas por el racismo estructural^50^. Estas reformulaciones del racismo en la biografía individual colectiva operan bajo métodos del “habitus colonial”^35^; por consiguiente, la internalización de los valores coloniales facilita la demostración del capital simbólico de la blancura. Estos recursos se activan a través de múltiples estrategias, impidiendo la movilidad social de las personas racializadas. Por eso, los proyectos de integración social, que “no ven la raza” o que procuran su “limpieza”, renuevan imaginarios y pactos que aseguran la transmisión o monopolio del capital simbólico europeo.

“El imaginario de la blancura, producido por el discurso de la limpieza de sangre, era una aspiración internalizada por muchos sectores de la sociedad colonial y actuaba como el eje alrededor del cual se construía la subjetividad […]. Ser “blancos” no tenía que ver tanto con el color de la piel, como con la escenificación personal de un imaginario cultural [...] por formas de producir y transmitir conocimientos. La ostentación de aquellas insignias culturales de distinción asociadas con el imaginario de blancura como signo de [...] transmisión de capital simbólico”.^27^

Grada Kilomba^27^, desde una perspectiva psicoanalítica, ofrece un panorama detallado de la herida psíquica generada por el colonialismo, en formas duraderas de racismo tácito o cotidiano. Según ella, el silenciamiento material impuesto a la boca-lengua negra configura el trauma colonial como parte de la historia en formas de “memorias de la plantación”. En su análisis, la violencia generada por la esclavitud, el colonialismo y el racismo cotidiano son la clave para comprender un trauma originado por la deshumanización de las personas negras, ostentando “en la combinación de narcisismo blanco y negación”, sin palabras para la simbolización de lo que se experimenta; y ese es un recurso extensivo de la plantación moderna colonial.

“Quiero usar la metáfora de la ‘plantación’ como símbolo de un pasado traumático que se vuelve a poner en escena a través del racismo cotidiano. Estoy hablando, por lo tanto, de un trauma colonial que ha sido memorizado. El pasado colonial es ‘memorizado’ en el sentido de que no ‘fue olvidado’. A veces uno preferiría no recordar, pero es incapaz de olvidar [...] la idea de una plantación es, por otro lado, un recordatorio de una historia colectiva de opresión racial, insultos, humillaciones y dolor, una historia que se anima en lo que denomino episodios del racismo cotidiano [...] este arreglo entre pasado y presente es capaz de retratar la sinrazón del racismo cotidiano como traumática”.^27^

La violencia racista se expresa en formas de mal trato que renuevan el trauma racial en el trabajo, la atención médica, la brutalidad policial, los perfilamientos raciales, la industria carcelaria, los patrones de belleza, los dispositivos del arte, la mediación intercultural, entre otros múltiples procedimientos marcados por microagresiones que activan heridas primarias que re-traumatizan a las personas racializadas. En los recursos destinados a la salud mental, suele expresarse en formas de omisión del padecimiento que generan dichas violencias y en la sobre medicalización de estos dolores. 

Esta herida asociada al racismo tiene que ver con memorias rotas, lo que conceptualizo aquí como “memorias del desamparo”, repartidas en la continuidad del colonialismo. Forman parte del despojo y la expropiación de las geografías afectivas comunitarias en cuerpos, tierras y territorios^51^, marcadas por la herida colonial expresada en emociones como rabia, vergüenza, soledad, desarraigo, miedo, abandono, asco, entre otras. Estas emociones son contextuales, en términos de la terapia narrativa^7^ quiere decir que evidencian cómo un valor importante se quiebra o se transgrede. Están asociadas a injusticias sistémicas que dejan huella, atestiguando el calado del mal trato, dejando un predominio de las historias dominantes que crean conclusiones o imágenes deterioradas de la identidad^52^. 

Un reto para los sistemas de atención en salud es lograr externalizar el malestar en función del racismo institucional. En otras palabras, se trata de comprender la doloridad que señala Vilma Piedade^53^, creada por los regímenes (internos, interpersonales, institucionales) que opacan las repercusiones del racismo en la salud. Se trata de acercarse a un dolor concreto vuelto silencio que inscribe a la negritud como la “carne más barata del mercado”^54^. 

Un dolor que los sistemas sanitarios no encajan en la doctrina del DSM-5. El sistema sanitario pretende no escuchar o entender. Colonialmente, “*no te entiendo*” funciona como procedimiento de clasificación racial que recuerda cuáles cuerpos son portadores de inteligibilidad y cuáles dejados en “desamparo”. Siglos atrás, la mezcla mulato con “tente en el aire” daba como resultado uno de los clasificadores raciales más desechables de la colonia: “*no te entiendo*”, un otro cada vez más distante del proyecto de civilización castizo. Esta función del habla colonial se retoma de la exploración en La Nueva Granada realizada por Santiago Castro^35^. 

El continuo de la doloridad, como parte del desamparo, se renueva en las tramas afectivas cotidianas. Convivir en las zonas de no-ser de Europa implica transitar la vida en una sobreexposición constante del no reconocimiento. Esta no se resuelve exclusivamente “con tener papeles”, aunque definitivamente es un paraguas necesario, se relaciona con traspasar las lógicas del multiculturalismo, la diversidad o la interculturalidad tan manoseados, dado que son disposiciones que poco alientan el agenciamiento, la autonomía o la interdependencia. 

Estar aquí supone contraste de memorias. El primer choque o aterrizaje virulento que contraponen los cuerpos racializados es que Europa no otorga pertenencia a quien no demuestra eficientemente la portabilidad de la *blanquitud.* En los términos que señalan Dent y Davis^55^, como propiedad que permite acceder a otros bienes. Se trata de contar con los medios de demostración locuaz -sin (neuro) divergencias- de que se poseen los patrimonios que facilitan los aprendizajes del desarrollo evolutivo o de progreso lineal que propone Europa. Siguiendo las teorías del aprendizaje de Piaget^56^, se trataría de evidenciar que la identidad migrante asimila, acomoda o equilibra la existencia a “la marca España”. Este ejercicio cognitivo de la congruencia con las pautas normativas del estado-nación está condicionado por el daltonismo racial^57^^)^ de una cultura política que recurre a la “invisibilidad de lo visible”^58^ o a la expresión “yo no veo colores” generando prácticas de negación o encubrimiento del racismo institucional. 

Los discursos prácticos asociados a “no ver colores” en salud, educación, empleo, cárcel, industria del arte, medios de comunicación, etc., implican que la “gestión de las migraciones” (como si fuésemos un ganado) se convierten en formas concretas de crueldad racista que buscan la docilidad o domesticación para “obtener los papeles”. 

Las disonancias afectivas arraigadas en la ley de extranjería marcan una existencia sin posibilidades de pertenencia. La voluntad o búsqueda de sentido de arraigo es perturbador, desgastante y movilizador, implica responder a múltiples demandas de gestión socioafectiva y administrativa que incrementan las heridas asociadas a la servidumbre del trato colonial.

“*Lo más difícil de vivir aquí es que nunca te sientes de aquí. El tiempo va pasando y todo lo que sucede aquí es para recordarte que no eres de aquí o que la vida no cuenta aquí. Yo, como mujer trabajadora del hogar y los cuidados, sé cada día lo que significa estar sin nada, vivir en el día a día, con la inseguridad de que en cualquier momento te pueden deportar o arrestar. Eso genera mucho miedo y hace que una no se atreva a denunciar o hablar de lo que nos pasa*”. (M. H. Entrevista en profundidad, 15 de abril de 2023)

Existe en la experiencia racializada migrante una imaginación proyectiva. Una capacidad inconmensurable de crear mundos otros, de vivir economías afectivas, por fuera del dominio blanco o entrando y saliendo de sus contornos. A continuación, profundizo en algunos de estos aspectos que bordean la sanación colectiva. 

### Imaginaciones para abrazar y cocer la lejanía de aquí y allá

“Cuando cocino me curo las penas. Se abriga un poco el corazón. Sofrío la nostalgia. Condimento la añoranza. Prendo fuego a la incertidumbre. Y remuevo poco a poco los sentires que habitan en lo profundo de mi alma. Cocinando vuelvo al cuerpo, a las raíces de mi tierra, a los aromas y sabores de mis abuelas cocinando ya no siento que hay un océano de por medio [...] Cuando preparo alimentos andinos A orillas del Mediterráneo que se funden en el fogón. Voy guisando a fuego lento Esta nueva identidad Este ‘ser’ y ‘estar’ En dos mundos a la vez Amando, soñando, extrañando, viviendo. Yo cocino para no olvidar. Escribo para no olvidar. Bailo para no olvidar que, aunque no soy ‘de aquí’. Esta Pacha me acoge. Me alimenta, me nutre. Y yo la habito a mi manera La honro y la agradezco Así, como me enseñaron mis abuelas”. (Anita Gutiérrez, poeta ecuatoriana en Valencia).

La posición resiliente antirracista de la poética de Anita funciona como fuego que transforma las condiciones dadas, son escenarios que transforman el dolor. No quiere decir ocultarlo o que se supera, sino que se convive creativamente. Esa posición re-existente permite desde la imaginación transfronteriza crear puentes de sostenimiento individual-colectivo. De este modo, se fragua una nueva espiritualidad u ontología a modo “luz desde lo oscuro”, en términos de Anzaldúa^59^, se crean nuevos territorios como *neplanta*, un lugar de “puente entre mundo”. 

Siguiendo la idea anterior, se trata de movimientos que entremezclan las tecnologías ancestrales con el activismo político conocido. Así la herida se transforma, porque trata de una comprensión de la justicia entre la “psicología y una mirada espiritual del activismo”^59^ para el desarrollo de una humanidad compasiva “con las manos en la razón del corazón”. Se trata de cosmogonías indígenas-afro, profundamente insurgentes, que migran con nosotres y desde ahí buscamos “corazonar”^60^ en Europa. Es difícil, pero lo estamos haciendo. De ahí que para muches se trata de afirmar que “migramos con nuestras raíces” y “nosotres no olvidamos”. Se confirma que estamos aquí con agencia propia:

*“Durante mucho tiempo nos acostumbramos a estar en los eventos, a participar de una manera en que no incomodamos, pero ya no es así. No basta con estar llevando la pancarta al principio de la mani, con que nos pongan en un cartel y usen nuestra imagen para decir que es un feminismo interseccional o antirracista. Nada de eso es suficiente, porque ahora estamos haciendo nuestra propia acción, estamos en ese momento”.* (K. M. Entrevista en profundidad, abril de 2024).

El concepto de justicia sanadora, en términos de atención al trauma racial, ha sido plenamente desarrollado por Erica Woodland y Cara Page^61^, como formas de transformación en coaliciones antirracistas. Estas autoras señalan tres principios claves para comprender la sanación antirracista decolonial: 1) el trauma es colectivo por tanto sana colectivamente; 2) no existe un modelo único para sanar; 3) y las estrategias de curación están arraigadas en el lugar y en tecnologías ancestrales. Esta reclamación de las prácticas ancestrales es sustantiva a cómo sanamos o habitamos la herida colonial. Se trata de aprendizajes anticoloniales arraigados en la transmisión de saberes o memorias que resisten al colonialismo. Actos que desde 1492 crean, desde la intimidad de la experiencia racial vivida, comunidad de afectos, palenques o quilombos en complicidad, vinculadas con biografías creadas en la “intimidad entre extraños”^62^. Diría supuestos extraños porque las coaliciones antirracistas justamente tratan del dolor común o compartido por una historia en el despojo que nos junta. 

Las personas racializadas provienen de memorias-territorios que expresan simultáneamente la paradoja de la aceptación y negación del proyecto colonizador. De este modo, las propuestas para la sanación no son uniformes, al contrario, prima la hibridez, no está exenta de divergencias, entre quienes procuran reformas e inciden en el estado, frente a los colectivos radicales que reniegan la participación dentro del proyecto de inclusión o integración que propone el estado español. De todos modos, en la experiencia racializada, la actuación política-laboral en B es parte de la coexistencia migra en comunalidad^63^. 

Las contradicciones o fricciones entre las propuestas de sanación colectiva se toman no como falta política, sino como parte del devenir en la racialización. No habitamos la pretendida pureza o congruencia blanca de los actos políticos. Queremos la riqueza que nos fue expropiada. Por eso, sanar la relación con el dinero hace parte de lo que buscamos, porque el legado de la abundancia se nos interrumpió y pasó a ser un producto del merecimiento o excelencia en los parámetros de la economía emocional colonial. Por eso estar aquí es contradictorio, porque buscamos esa “devolución del oro”; de nuestro estatuto en el “vivir sabroso” o bien vivir que nos fue arrebatado; mientras que Europa somete ese deseo a la intemperie o al olvido selectivo. Más aún, el placer o celebración de la hispanidad es un recuerdo fundacional de nuestro dolor; de cómo somos devueltos a este lugar del no tener. ¡Eso da rabia! Más aún, que se pretenda que rechacemos el dinero porque es malo o por “amor al arte”, un arte que nos fue expropiado da furia. 

En España, la memoria histórica refiere al franquismo, no al colonialismo. Igual que holocausto para Alemania refiere a los judíos no al maafa de la trata transatlántica que pare a Europa. Eso también monta en cólera. Distancia nuestros sentidos y nos da la valentía para reclamar lo que nos pertenece aquí: “Esta tierra también es nuestra”. 

Provenimos de tierras con múltiples caminos en la generación de revoluciones o hazañas históricas inimaginables como la haitiana^64^. Provenimos del lenguaje de los altares, los textiles y las estéticas, palabras o idiomas preservados o reinventados en el trayecto, la ideación de innumerables tecnologías espirituales para atesorar cosmogonías otras del mundo. Tenemos formas de siembra-cosecha, arquitecturas, medicinas, literaturas, música, danza, alimentos, etc., no traducibles, obras cotidianas invaluables asociadas con la reinvención futurista del mundo. Buscamos refugio “casa adentro” (expresión del pueblo afroecuatoriano). Aquí reinterpreto el refugio, en el sentido de amparo o red que crean las propias comunidades migrantes racializadas al margen del anti-refugio que plantean las políticas de acogida del estado español. 

En el trayecto antirracista, como plantea el Colectivo Ayllu^65^, necesitamos “escupir la rabia” como parte de “narrativas inapropiadas” que incomodan a la blanquitud. Siguiendo a Lugones^66^, la rabia trata de una emoción proscrita en términos de la convención civilizatoria. Les cuerpos-comunidades racializades están tremendamente estigmatizados, por eso, sentir la ira es un atributo que no está concedido. En este sentido, acceder a ella permite la creación de límites y la apertura de relaciones no igualitarias, pero sí respetuosas. 

El cajón de herramientas comunal, como en el flamenco “de ida y vuelta”, es anticolonial. Está activo para sostener las vidas racializadas. Se trata de activaciones ancestrales que viajan a través del tiempo en archivos intangibles de la memoria. Se trata de poéticas de afirmación que renombran el dolor, por fuera del síndrome, desde lugares estéticos que crean sanación, más allá de la frialdad de los sistemas de citas o controles sanitarios en Madrid. Cuando les trans irónicamente dicen: “*nuestra venganza es ser bonitas*”, “*la alegría es mi venganza*”, o “*escupir la rabia para sanar*”, etc., muestran ese talento de acuerpar por fuera de la lógica cis, como “aves errantes transtemporaneas e indestructibles”^44^^,^^67^^)^

Audre Lorde^68^ enfatiza que la rabia hay que explicitarla, reconocer su fuerza, porque permite trazar los límites frente al racismo. Para ella, es una fuente creadora para la liberación y advierte “cuando le damos la espalda a la ira también se la damos al conocimiento”, porque se actúa desde la corrección política. La rabia permite atravesar el egoconquiro^69^, es decir, es una emoción que interpela la razón blanca europea, de una subjetividad forjada en la diferencia colonial.

La rabia es un registro (no) permitido en la otredad, porque daría cuenta de su agencia. María Lugones lo explica del siguiente modo: “enojándose, las y los subordinados señalan que se han tomado a sí mismas/os en serio; consideran que tienen la capacidad, y el derecho, de ser jueces de quienes los rodean”^66^. Para ella es una emoción política que da cuenta de la resistencia. La cuestión sustancial es, según Anzaldúa^59^, cómo hacer para que su energía no nos destruya “entre pares”. Hay una cuestión de latencia destructiva, disruptiva, en la rabia contenida por los silencios que, mantenida en el tiempo, destruye el propio cuerpo. Como señalan las preguntas retadoras de Lorde^70^: “¿Qué palabras no tienes todavía para explicar el sufrimiento? ¿Qué necesitas decir? ¿Qué tiranías te tragas día a día e intentas hacer tuyas, hasta que te enfermen y te maten?”. Ella vincula la ira con las “tiranías atragantadas” que necesitan ser liberadas del miedo. De vuelta a Lugones^66^, se trata de usar la ira desbordante para sobrepasar la respetabilidad aprendida y decir sin destruir(nos), como comunidades permeadas por la rabia, y su reverso la tristeza, engendradas en el miedo. 

Develar la amnesia colonial que caracteriza a Europa abre la rabia. Retomo este concepto de pérdida de la memoria del trabajo cinematográfico realizado por Claudia Claremi^71^, quien visibiliza cómo la sociedad española, a través de una ceremonia típica, las fiestas de Alcoy, caricaturiza la crueldad que implica la encarnación negra como “pieles blancas con máscaras negras”. Se tratan de tecnologías del *black face* que se validan bajo argumentos de la cultura popular, pero que reflejan el profundo calado del racismo simbólico en la sociedad. 

Sanar colectivamente es un recurso que nos permite imaginar soluciones, mundos propios con la definición, escenificación e intensidad de nuestro poder. Es decir, nuestra rabia antirracista ha permitido crear autonomía cimarrona, es decir, el talento de hacer un cerco en el que la blanquitud no entra. Eso, en sí mismo, da miedo, pero consolida la voz-escucha en modo que afianzan los territorios de la imaginación antirracista, que sobrepasan la moral de la respetabilidad, anclados en la diferencia colonial. 

La búsqueda de coreografías propias es una práctica que el movimiento migrante gesta desde inicios de la década de 1980^72^; sin embargo, es a partir de 2015 aproximadamente que se inician las creaciones de coaliciones más formales entre comunidades racializadas. Esos espacios de encuentro, para compartir herramientas de entendimiento y *fugitividad,* están abriendo agujeros a las nociones tradicionales de la migración, estos elementos en curso dan cuenta de un escenario político distintivo de la agencia migrante racializada en el entorno madrileño.

## CONCLUSIÓN ABIERTA

A modo de conclusión abierta, es necesario el reconocimiento de la herida colonial y de la actualidad de los sistemas coloniales-racistas por parte del sector sanitario. La acción colectiva y el vínculo con el territorio, desde un análisis colonial y estructural del racismo, permiten una perspectiva crítica con el sistema eurocentrado de salud mental, tanto a nivel epistemológico como práctico. Por ello, se propone profundizar acerca de cómo los escenarios de la salud pública requieren de políticas, programas, servicios que atiendan de manera diferencial los asuntos que atañen al trauma racial y duelo migratorio, desde abordajes históricos y narrativos que posibiliten la imaginación radical antirracista, abriendo espacio a la sanación desde perspectivas que contribuyan a la reparación histórica económica o material-simbólica de la herida colonial.
